# Supplementary material for: From sequence to enzyme mechanism using multi-label machine learning
Source: BMC Bioinformatics. 2014 May 19;15:150. doi: 10.1186/1471-2105-15-150 (PMC4229970; doi:10.1186/1471-2105-15-150)
Supplement: Additional file 2 — Java code of ml2db. Additional file ml2db_code.tar.gz contains the Java source code to run the multi-label machine learning experiments and save the results to database. The code’s Javadoc is included. [file 1471-2105-15-150-S2.zip › additional file 2/ml2db/utils/doc/index-files/index-7.html]

G-Index


JavaScript is disabled on your browser.


- Overview
- Package
- Class
- Use
- Tree
- Deprecated
- Index
- Help

- Prev Letter
- Next Letter

- Frames
- No Frames

- All Classes

A B C D E F G H I J K L M N O P Q R S T U V W X Y 


## G

gaussian() - Method in class edu.cornell.lassp.houle.RngPack.RandomElement
:   gaussian() uses the Box-Muller algorithm to transform raw()'s into
    gaussian deviates.

gaussian(double) - Method in class edu.cornell.lassp.houle.RngPack.RandomElement


generateAKeyValues(T) - Method in class uk.ac.standrews.utils.main.maputils.OneToManyMap


generateInternalKeys() - Method in class uk.ac.standrews.utils.main.maputils.MapOfMaps


generateSupersets() - Method in class uk.ac.standrews.utils.main.collectionutils.setutils.SupersetsManager


generateSupersets() - Method in class uk.ac.standrews.utils.main.setutils.SupersetsManager


getAddedEnd() - Method in class uk.ac.standrews.utils.main.diff.Difference
:   The point at which the addition ends, if any.

getAddedStart() - Method in class uk.ac.standrews.utils.main.diff.Difference
:   The point at which the addition starts, if any.

getAKeyValues(T) - Method in class uk.ac.standrews.utils.main.maputils.OneToManyMap
:   Get the set of unique values for this key.

getAllMatchedSubstring(String, String, boolean) - Static method in class uk.ac.standrews.utils.main.RegExpUtils
:   Returns all regular expression matches in the text (as strings)

getAllMatchedSubstringsEndpoints(String, String, boolean) - Static method in class uk.ac.standrews.utils.main.RegExpUtils
:   Gets the start and end index of all regular expression matches in the
    text

getAttributeValue(String) - Method in class uk.ac.standrews.utils.main.webutils.simpledomparser.XmlNode
:   Get the attribute value for the given attribute name.

getBasicTypeOfDataColumn(ArrayList<String>) - Static method in class uk.ac.standrews.utils.main.database.DbUtils
:   Given a list of strings, it returns a generic data type for the database
    column that could best contain them: INTEGER, DOUBLE, STRING
    If the strings passes a parseInteger: type = INTEGER if the strings
    contain digits, 'E' (for exponential), dot, plus, minus: type = DOUBLE If
    the strings contain letters or other characters: type = STRING Note: If
    the string contains only digits, but also intervening white spaces: type
    = STRING

getCanonicalVersion(boolean[]) - Method in class uk.ac.standrews.utils.main.BooleanArrayPool


getCanonicalVersion(String) - Method in class uk.ac.standrews.utils.main.stringutils.StringPool


getChildElements() - Method in class uk.ac.standrews.utils.main.webutils.simpledomparser.XmlNode
:   public Object[] getChildElements() { return m\_childElements.toArray(); }

getChosenOption() - Method in class uk.ac.standrews.utils.main.guiutils.SimpleRadioButtonPanel


getCollection() - Static method in class uk.ac.standrews.utils.test.CollectionUtilsTest


getColumn2ValueMap() - Method in class uk.ac.standrews.utils.main.database.TableRow
:   Returns a comma separated string of the values

getColumnName() - Method in class uk.ac.standrews.utils.main.database.TableColumn
:   Gets the column name

getColumnNames() - Method in class uk.ac.standrews.utils.main.database.Table
:   Gets column names for the table

getColumns() - Method in class uk.ac.standrews.utils.main.database.Table
:   Get the `TableColumn` list

getColumns() - Method in class uk.ac.standrews.utils.main.database.TableRow


getColumnsfrom2DArray(String[][]) - Static method in class uk.ac.standrews.utils.main.database.DbUtils
:   Takes a 2 dimensional array of data and returns the columns (as an array
    list of array lists)

getColumnsValues(ResultSet, int, int, int, int, int, int, String, boolean, String) - Method in class uk.ac.standrews.utils.main.database.DbReader
:   Get three columns of a `ResultSet` and store them in a [external
    key, internal key, value] map

getColumnValues(ResultSet, int, AbstractCollection<String>, int) - Method in class uk.ac.standrews.utils.main.database.DbReader
:   Get the nth column of a `ResultSet`

getCommonElements(int[], int[]) - Static method in class uk.ac.standrews.utils.main.collectionutils.ArrayUtils
:   Gets the elements present in both lists

getCommonElements(Object[], Object[]) - Static method in class uk.ac.standrews.utils.main.collectionutils.ArrayUtils
:   Gets the elements present in both lists

getCommonElements(ArrayList, ArrayList) - Static method in class uk.ac.standrews.utils.main.collectionutils.ListUtils
:   Gets the elements present in both lists

getConnection() - Method in class uk.ac.standrews.utils.main.database.DbConn


getConnection(String) - Static method in class uk.ac.standrews.utils.main.database.DbConnectionSingleton


getConnection() - Method in class uk.ac.standrews.utils.test.database.DbUtilsTest


getConnectionParametersString() - Method in class uk.ac.standrews.utils.main.database.DbConn
:   Get the connection log (database driver + url)

getConnectionPropertiesFilePath() - Method in class uk.ac.standrews.utils.main.database.DbConn


getCreator() - Static method in class uk.ac.standrews.utils.test.database.TableCreatorTest


getDatabaseName() - Method in class uk.ac.standrews.utils.main.database.DbConn
:   Gets the database the connection is connected to

getDatabaseName() - Method in class uk.ac.standrews.utils.main.database.DbManager


getDatabaseType() - Method in class uk.ac.standrews.utils.main.database.DbConn


getDataDbManager() - Static method in class uk.ac.standrews.utils.test.database.DbManagerTest


getDataType() - Method in class uk.ac.standrews.utils.main.database.TableColumn
:   Gets the column data type

getDataTypeForAnArray(ArrayList<String>) - Static method in class uk.ac.standrews.utils.main.database.DbUtils
:   Guesses from data type and length the appropriate SQL type of a column to
    contain the data in the array

getDateForFileName() - Static method in class uk.ac.standrews.utils.main.TimeUtils
:   getFileTimeStamp returns a date stamp that can be attached to file names

getDateHourStampForFileName() - Static method in class uk.ac.standrews.utils.main.TimeUtils
:   Date-time stamp (up to hours) that can be attached to file names

getDateStamp() - Static method in class uk.ac.standrews.utils.main.TimeUtils
:   Returns a date stamp in the format: 20070117 yyyymmdd

getDateStrings() - Static method in class uk.ac.standrews.utils.main.TimeUtils
:   getDateStrings()

getDateTimeMillisecondsStamp() - Static method in class uk.ac.standrews.utils.main.TimeUtils
:   Returns a date-time-milliseconds stamp in the format: 20070117\_111847\_123
    yyyymmdd\_hhmmss\_lll

getDateTimeNanoSecondsStamp() - Static method in class uk.ac.standrews.utils.main.TimeUtils
:   Returns a date-time-milliseconds-nanoseconds stamp in the format:
    20070117\_111847\_123\_3000 yyyymmdd\_hhmmss\_lll\_nnnn (the n digits are not
    the nanoseconds! just a progressive stamp)

getDateTimeStamp() - Static method in class uk.ac.standrews.utils.main.TimeUtils
:   Returns a date-time stamp in the format: 20070117\_111847 yyyymmdd\_hhmmss

getDbConnection() - Method in class uk.ac.standrews.utils.main.database.DbManaged


getDbConnection() - Method in class uk.ac.standrews.utils.main.database.DbManager


getDbConnProps() - Static method in class uk.ac.standrews.utils.test.database.DbConnPropsTest


getDbCreator() - Method in class uk.ac.standrews.utils.main.database.DbManager


getDbCreator() - Static method in class uk.ac.standrews.utils.test.database.DbCreatorTest


getDbManager() - Method in class uk.ac.standrews.utils.main.database.DbManaged


getDbManager() - Method in class uk.ac.standrews.utils.main.database.Table
:   public int saveRowToTable(TableRow row) {
    // write row to table
    TableWriter writer = this.getTableWriter();
    int resultId = writer.insertReturnsKey(row);
    if (m\_debug ) {
    System.out.println("Written row with id: "
    + resultId
    + " to table "
    + this.getTableName()
    + " database "
    + this.getDbManager().getDatabaseName() + " - "
    + this.getClass().getName());
    }
    return resultId;
    }

getDbMetaData() - Method in class uk.ac.standrews.utils.main.database.DbReader


getDbReader() - Method in class uk.ac.standrews.utils.main.database.DbManaged


getDbReader() - Method in class uk.ac.standrews.utils.main.database.DbManager


getDbReader() - Static method in class uk.ac.standrews.utils.test.database.DbReaderTest


getDbWriter() - Method in class uk.ac.standrews.utils.main.database.DbManager


getDbWriter() - Static method in class uk.ac.standrews.utils.test.database.DbWriterTest


getDeletedEnd() - Method in class uk.ac.standrews.utils.main.diff.Difference
:   The point at which the deletion ends, if any.

getDeletedStart() - Method in class uk.ac.standrews.utils.main.diff.Difference
:   The point at which the deletion starts, if any.

getDirectoryFiles(String) - Static method in class uk.ac.standrews.utils.main.FileUtils
:   Gets the list of files in a directory From
    http://www.faqs.org/docs/javap/c10/s2.html

getDirectoryPath(String) - Static method in class uk.ac.standrews.utils.main.FileUtils
:   Get the parent of a full file path (directory + filename)

getDoubleFromHashmap(HashMap, String) - Static method in class uk.ac.standrews.utils.main.maputils.MapUtils
:   Gets a double from a hashMap

getDoubleIntervals(Double, Double, Double) - Static method in class uk.ac.standrews.utils.main.Utils


getDriverName() - Method in class uk.ac.standrews.utils.main.database.DbConn


getElapsedTime(long, long) - Static method in class uk.ac.standrews.utils.main.TimeUtils
:   Returns a string with the elapsed time in a hour:min:sec.msec format

getElements() - Method in class uk.ac.standrews.utils.main.collectionutils.setutils.Set


getElements() - Method in class uk.ac.standrews.utils.main.setutils.Set


getElementsSize() - Method in class uk.ac.standrews.utils.main.collectionutils.setutils.Set


getElementsSize() - Method in class uk.ac.standrews.utils.main.setutils.Set


getEndTimeColumn() - Static method in class uk.ac.standrews.utils.main.database.DbUtils
:   A table column to store a timestamp for the end of an experiment

getESummaryXML(String, String) - Static method in class uk.ac.standrews.utils.main.EntrezUtils
:   Gets an XML tree of elements (SimpleElement) from eSummary Entrez
    service.

getExternalInternalKeyMap() - Method in class uk.ac.standrews.utils.main.maputils.MapOfMaps
:   A one to many map containing for each external key the list of unique
    internal keys in its map.

getExtStringPool() - Method in class uk.ac.standrews.utils.main.maputils.MapOfMaps


getFileNameFromDirectory(String, String) - Static method in class uk.ac.standrews.utils.main.FileUtils
:   Get the first file name in the directory matching the given pattern
    string.

getFileNameFromFullPath(String) - Static method in class uk.ac.standrews.utils.main.FileUtils
:   Get the filename from a full file path (directory + filename)

getFileSizeInKb(String) - Static method in class uk.ac.standrews.utils.main.FileUtils
:   Get the file size in Kb

getFirstMatchedSubstring(String, String) - Static method in class uk.ac.standrews.utils.main.RegExpUtils
:   Gets the first matched substring in the text (dot all mode disabled > the
    dot does not match the \n character)

getFirstMatchedSubstring(String, String, boolean) - Static method in class uk.ac.standrews.utils.main.RegExpUtils
:   Gets the first matched substring in the text

getFirstValueOccurrence(Map<String, String>, String) - Static method in class uk.ac.standrews.utils.main.maputils.MapUtils
:   Get all the keys that have the given value in a Map\

getFlag(String, String[]) - Static method in class uk.ac.standrews.utils.main.guiutils.OptionUtils


getFlag(String, String[]) - Static method in class uk.ac.standrews.utils.main.Utils


getFrequencies() - Static method in class uk.ac.standrews.utils.test.stats.StatUtilsTest


getGunzipInputStream(BufferedInputStream) - Static method in class uk.ac.standrews.utils.main.FileUtils


getHighestValue(TreeSet<Double>) - Static method in class uk.ac.standrews.utils.main.collectionutils.setutils.SetUtils


getHighestValue(TreeSet<Double>) - Static method in class uk.ac.standrews.utils.main.setutils.SetUtils


getHighestValuedKey(TreeMap<String, Double>) - Static method in class uk.ac.standrews.utils.main.maputils.MapUtils
:   Return the key with the highest value (or the first key with the highest
    value, if many have the same value).

getHighestValuedKeyRandomly(TreeMap<String, Double>) - Static method in class uk.ac.standrews.utils.main.maputils.MapUtils
:   Return the key with the highest value (or one key \*at random\* among the
    ones with the highest value).

getHost() - Method in class uk.ac.standrews.utils.main.database.DbConn


getHourOnlyStamp() - Static method in class uk.ac.standrews.utils.main.TimeUtils
:   Returns a time stamp in the format (for 11 am, 18 min, 47 sec): 11 hh

getHumanReadableTimeStamp() - Static method in class uk.ac.standrews.utils.main.TimeUtils
:   Returns a human readable date-time stamp

getHumanReadableTimeStampMillisec() - Static method in class uk.ac.standrews.utils.main.TimeUtils


getId() - Method in class uk.ac.standrews.utils.main.collectionutils.setutils.Set


getId() - Method in class uk.ac.standrews.utils.main.setutils.Set


getIdColumn() - Static method in class uk.ac.standrews.utils.main.database.DbUtils
:   get the definition for an "id" column of type auto-increment (which is
    primary key)

getIncrements(Object, Object, Double) - Static method in class uk.ac.standrews.utils.main.Utils
:   Returns a vector of numbers, starting from a minimum value, with
    incremental increases until the maximum value.

getIncrementsFromRange(Integer, Double) - Static method in class uk.ac.standrews.utils.main.Utils
:   Returns a set of increments.

getIndexedMap(Object[]) - Static method in class uk.ac.standrews.utils.main.maputils.MapUtils
:   Get a map with entries composed of: an integer id as key and an entry as
    value

getInsertSql(TableRow) - Method in class uk.ac.standrews.utils.main.database.TableWriter
:   Returns the sql "insert into" statement for the row

getInstance(String) - Static method in class uk.ac.standrews.utils.main.database.DbManagerSingleton


getInstance(String) - Static method in class uk.ac.standrews.utils.main.LoggerCreator


getInstance(double, double, int, int, int) - Static method in class uk.ac.standrews.utils.main.stats.PseudoTruncatedParetoSingleton


getInstance() - Static method in class uk.ac.standrews.utils.main.stats.UniformRandomSingleton


getIntegerIntervals(Integer, Integer, Double) - Static method in class uk.ac.standrews.utils.main.Utils
:   Returns a vector of numbers, starting from a minimum value, with
    incremental increases until the maximum value.

getInternalBlocks(String, XmlNode, File) - Static method in class uk.ac.standrews.utils.main.webutils.simpledomparser.XmlSearcher
:   Gets the children of a given xml tag

getInternalKeys() - Method in class uk.ac.standrews.utils.main.maputils.MapOfMaps


getIntStringPool() - Method in class uk.ac.standrews.utils.main.maputils.MapOfMaps


getJavaSystemProperties() - Static method in class uk.ac.standrews.utils.main.PropertiesUtils
:   Gets the Java system properties

getJavaSystemProperties() - Static method in class uk.ac.standrews.utils.main.Utils
:   Gets the Java system properties

getKeys() - Method in class uk.ac.standrews.utils.main.maputils.TableMap


getKeysStrings(Properties) - Static method in class uk.ac.standrews.utils.main.PropertiesUtils
:   Get the properties keys as strings

getKeysVectorValuesVector() - Method in class uk.ac.standrews.utils.main.maputils.OneToManyMap
:   Get 1.

getKeysWithValue(U) - Method in class uk.ac.standrews.utils.main.maputils.OneToManyMap
:   Returns all the keys having a certain value

getKeyValuePairSize() - Method in class uk.ac.standrews.utils.main.maputils.TableMap
:   Get the number of key-value pairs in the map

getKeyValues(String) - Method in class uk.ac.standrews.utils.main.maputils.TableMap


getKeyValuesPairsSize() - Method in class uk.ac.standrews.utils.main.maputils.OneToManyMap
:   Get the number of unique key-value pairs in the map.

getListOfDbTables() - Method in class uk.ac.standrews.utils.main.database.DbReader
:   Gets the list of tables for a database

getListOfValuesString(Vector<String>, String) - Static method in class uk.ac.standrews.utils.main.database.DbUtils
:   Transforms a list of values into a comma separated list

getLoadDataInfileSql(String, String, String, int, String) - Method in class uk.ac.standrews.utils.main.database.TableWriter


getLongestCommonSubsequences() - Method in class uk.ac.standrews.utils.main.diff.Diff
:   Returns an array of the longest common subsequences.

getLongestStringLength(ArrayList<String>) - Static method in class uk.ac.standrews.utils.main.collectionutils.ListUtils
:   Get the length of the longest string in an array

getManager() - Method in class uk.ac.standrews.utils.main.database.Managed


getManager() - Static method in class uk.ac.standrews.utils.test.setutils.SupersetsManagerTest


getManager2() - Static method in class uk.ac.standrews.utils.test.setutils.SupersetsManagerTest


getManager3() - Static method in class uk.ac.standrews.utils.test.setutils.SupersetsManagerTest


getMap(String) - Method in class uk.ac.standrews.utils.main.database.DbReader
:   Converts a query resultset into a map.

getMap(String, int, String, boolean, String) - Method in class uk.ac.standrews.utils.main.database.DbReader
:   Converts a query resultset into a map.

getMap() - Method in class uk.ac.standrews.utils.main.maputils.TableMap
:   Get the keys to values map

getMap() - Static method in class uk.ac.standrews.utils.test.maputils.MapUtilsTest


getMap1(boolean) - Static method in class uk.ac.standrews.utils.test.maputils.MapOfMapsTest


getMap1() - Static method in class uk.ac.standrews.utils.test.maputils.MapUtilsTest


getMap2() - Static method in class uk.ac.standrews.utils.test.maputils.MapUtilsTest


getMatcher(String, String, boolean) - Static method in class uk.ac.standrews.utils.main.RegExpUtils
:   Get a matcher trained on the given pattern/regular expression and
    pointing at the text of interest.

getMilliseconds() - Static method in class uk.ac.standrews.utils.main.TimeUtils
:   Returns a milliseconds time stamp

getMillisecTimeStampForFileName() - Static method in class uk.ac.standrews.utils.main.TimeUtils
:   Date-time stamp (up to milliseconds) that can be attached to file names

getNameForEntrezGeneId(String) - Static method in class uk.ac.standrews.utils.main.EntrezUtils
:   Get the entrez gene name for an entrez gene id

getNanoSecondsStamp() - Static method in class uk.ac.standrews.utils.main.TimeUtils
:   Returns a nanoseconds stamp (it's a progressive stamp actually, not the
    actual nanoseconds in desktop-clock style!)

getNextNanoSecondsStamp(String) - Static method in class uk.ac.standrews.utils.main.TimeUtils
:   Returns a nanoseconds stamp + 1 (it's a progressive stamp actually, not
    the actual nanoseconds in desktop-clock style!)

getNonEmptyKeys() - Method in class uk.ac.standrews.utils.main.maputils.OneToManyMap
:   Get the set of unique, non-empty-string keys for this map.

getNonEmptyKeyValuesPairsSize() - Method in class uk.ac.standrews.utils.main.maputils.OneToManyMap
:   Get the number of unique, non-empty key-value pairs in the map.

getNonEmptyValues() - Method in class uk.ac.standrews.utils.main.maputils.OneToManyMap
:   Get the set of unique, non-empty-string values for this map.

getNoOfRows() - Method in class uk.ac.standrews.utils.main.database.TableColumn
:   Gets the number of rows in the column

getNumberedXmlTree() - Static method in class uk.ac.standrews.utils.test.XmlNodeTest


getNumberOfMatchedSubstrings(String, String, boolean) - Static method in class uk.ac.standrews.utils.main.RegExpUtils
:   Counts the number of regular expression matches in the text

getOffset() - Method in class uk.ac.standrews.utils.main.stats.TruncatedPareto


getOption(String, String[]) - Static method in class uk.ac.standrews.utils.main.guiutils.OptionUtils
:   GetOption(String opt, String[] options) gets an option value out of a
    list of options

getOption(String, String[]) - Static method in class uk.ac.standrews.utils.main.Utils
:   getOption(String opt, String[] options) gets an option value out of a
    list of options

getPareto() - Method in class uk.ac.standrews.utils.main.stats.TruncatedPareto


getParetoFrequencies(int) - Method in class uk.ac.standrews.utils.main.stats.TruncatedPareto
:   Extracts numbers from a pareto distribution for the given number of times
    and records the freqencies with which the integers have been extracted

getPassword() - Method in class uk.ac.standrews.utils.main.database.DbConn


getPdbSimpleElement() - Static method in class uk.ac.standrews.utils.test.XmlNodeTest


getPdbXmlReader() - Static method in class uk.ac.standrews.utils.test.XmlUtilsTest


getPort() - Method in class uk.ac.standrews.utils.main.database.DbConn


getPotentialDirectory(File) - Static method in class uk.ac.standrews.utils.main.FileUtils
:   Get a directory out of a path

getPseudoRandomNumbers() - Method in class uk.ac.standrews.utils.main.stats.PseudoTruncatedPareto


getPseudoTruncatedPareto() - Method in class uk.ac.standrews.utils.main.stats.PseudoTruncatedParetoSingleton


getPubMedUrl(String) - Static method in class uk.ac.standrews.utils.main.EntrezUtils
:   Generates pubMed URL adding pubMed ids (as list separated by commas)

getRandomInt(int) - Static method in class uk.ac.standrews.utils.main.stats.StatUtils
:   Problem: generates a new Random at each call...

getRandomUtils() - Static method in class uk.ac.standrews.utils.test.stats.RandomUtilsTest


getRandomValue(T) - Method in class uk.ac.standrews.utils.main.maputils.OneToManyMap
:   Get randomly one of the values of this key

getReader() - Static method in class uk.ac.standrews.utils.test.database.TableReaderTest


getReaderFromInputStream(InputStream) - Static method in class uk.ac.standrews.utils.main.webutils.WebUtils
:   Creates a buffered reader from an input stream

getResultSet(String, DbConn) - Static method in class uk.ac.standrews.utils.main.database.DbUtils
:   Executes an sql query returning a result set

getResultSetColumnAsVector(ResultSet, int) - Static method in class uk.ac.standrews.utils.main.database.DbUtils
:   Takes a resultSet and returns an array of Strings

getResultSetNumberOfColumns(ResultSet) - Static method in class uk.ac.standrews.utils.main.database.ResultSetUtils


getReverseMap() - Method in class uk.ac.standrews.utils.main.maputils.TableMap
:   Get the values to keys map

getRowKo() - Static method in class uk.ac.standrews.utils.test.database.TableRowTest


getRowOk() - Static method in class uk.ac.standrews.utils.test.database.TableRowTest


getRowValues(ResultSet, int) - Method in class uk.ac.standrews.utils.main.database.TableReader
:   Get the nth row of a `ResultSet`

getSeed() - Method in class edu.cornell.lassp.houle.RngPack.Ranecu


getSelectStarSql(String) - Static method in class uk.ac.standrews.utils.main.database.SqlUtils
:   Generates a select \* query text (eg.

getSelectWhereEqualSql(String, String, String) - Static method in class uk.ac.standrews.utils.main.database.SqlUtils
:   gets sql code for a select on one column of a table, with a where x = y
    condition specific for that column

getSelectWhereEqualSql(String, String, String, String, String) - Static method in class uk.ac.standrews.utils.main.database.SqlUtils
:   Select on two columns of a table, with two "where" conditions, , one for
    each provided column

getSelectWhereLikeSql(String, String, String) - Static method in class uk.ac.standrews.utils.main.database.SqlUtils
:   Gets sql code for a select on one column of a table, with a where x like
    y condition specific for that column

getSelectWhereSql(String, String, String) - Static method in class uk.ac.standrews.utils.main.database.SqlUtils
:   gets sql code for a select on one column of a table, with a where
    condition

getSeparator() - Method in class uk.ac.standrews.utils.main.collectionutils.setutils.SupersetsManager


getSeparator() - Method in class uk.ac.standrews.utils.main.setutils.SupersetsManager


getSerialNumericalEnding(String, String) - Static method in class uk.ac.standrews.utils.main.stringutils.StringUtils
:   Get a progressive +1 numerical ending.

getSet(String, TreeSet<String>) - Static method in class uk.ac.standrews.utils.test.setutils.SetTest


getSetA() - Static method in class uk.ac.standrews.utils.test.setutils.SetTest


getSetAB() - Static method in class uk.ac.standrews.utils.test.setutils.SetTest


getSetABC() - Static method in class uk.ac.standrews.utils.test.setutils.SetTest


getSetAC() - Static method in class uk.ac.standrews.utils.test.setutils.SetTest


getSetAD() - Static method in class uk.ac.standrews.utils.test.setutils.SetTest


getSets() - Method in class uk.ac.standrews.utils.main.collectionutils.setutils.SupersetsManager


getSets() - Method in class uk.ac.standrews.utils.main.setutils.SupersetsManager


getShortestStringLength(ArrayList<String>) - Static method in class uk.ac.standrews.utils.main.collectionutils.ListUtils
:   Get the length of the shortest string in an array

getSimpleSelectSql(String, String) - Static method in class uk.ac.standrews.utils.main.database.SqlUtils
:   gets sql code for a select on one column of a table

getSimpleSerie(Double, Double, int) - Static method in class uk.ac.standrews.utils.main.Utils
:   Gets a series of doubles from a min incrementing by a given increment for
    a number of iterations

getSize() - Method in class uk.ac.standrews.utils.main.stringutils.Pool


getSqlForSelectLike(String, String, String, String) - Static method in class uk.ac.standrews.utils.main.database.SqlUtils
:   Sql code for a select "LIKE"

getSqlSelectByIdByRegExp(String, String, String, String, String) - Static method in class uk.ac.standrews.utils.main.database.SqlUtils
:   Generates the sql command for a select of a value corresponding to a
    certain id AND matching a certain regex string

getSqlSelectByRegExp(String, String, String) - Static method in class uk.ac.standrews.utils.main.database.SqlUtils
:   Generates the sql command for a select matching a certain regex string

getSqlTimestamp() - Static method in class uk.ac.standrews.utils.main.TimeUtils
:   Gets a sql.Timestamp object of the current time (precise to the second:
    nanoseconds are set to zero)

getSqlTimestampString() - Static method in class uk.ac.standrews.utils.main.TimeUtils


getSqlToCreateTable() - Method in class uk.ac.standrews.utils.main.database.TableCreator
:   Get the SQL statement to create the table

getSqlToDropTable(String) - Method in class uk.ac.standrews.utils.main.database.DbCreator
:   Get sql command to drop table

getSqlToDropTable(String) - Static method in class uk.ac.standrews.utils.main.database.SqlUtils
:   Get sql to drop table

getStartTimeColumn() - Static method in class uk.ac.standrews.utils.main.database.DbUtils
:   A table column to store a timestamp for the beginning of an experiment

getStatement() - Method in class uk.ac.standrews.utils.main.database.DbConn
:   Gets a statement from a new connection

getStatement() - Method in class uk.ac.standrews.utils.main.database.DbReader


getStreamFromFileUri(String) - Static method in class uk.ac.standrews.utils.main.webutils.WebUtils


getStreamFromGzipUrl(String) - Static method in class uk.ac.standrews.utils.main.webutils.WebUtils


getStreamFromURL(String) - Static method in class uk.ac.standrews.utils.main.webutils.WebUtils
:   Creates an input stream from a URL (gets all the HTML or XML contained in
    the page)

getString(double[]) - Static method in class uk.ac.standrews.utils.main.collectionutils.ArrayUtils
:   Returns a string representation for an array

getString(int[]) - Static method in class uk.ac.standrews.utils.main.collectionutils.ArrayUtils
:   Returns a string representation for an array

getString(String[]) - Static method in class uk.ac.standrews.utils.main.collectionutils.ArrayUtils
:   Returns a string representation for an array

getStringDoubleMap() - Static method in class uk.ac.standrews.utils.test.maputils.MapUtilsTest


getStringFromBufferedReader(BufferedReader) - Static method in class uk.ac.standrews.utils.main.FileUtils


getStringFromBufferedReader(BufferedReader) - Static method in class uk.ac.standrews.utils.main.webutils.WebUtils
:   Get a string from a buffered reader

getStringFromFile(String) - Static method in class uk.ac.standrews.utils.main.FileUtils
:   Get all the text in a file as a single string

getStringFromUrl(String, boolean) - Static method in class uk.ac.standrews.utils.main.webutils.WebUtils
:   Get a string from a URL

getSubsets() - Method in class uk.ac.standrews.utils.main.collectionutils.setutils.Set


getSubsets() - Method in class uk.ac.standrews.utils.main.setutils.Set


getSubsetsSize() - Method in class uk.ac.standrews.utils.main.collectionutils.setutils.Set


getSubsetsSize() - Method in class uk.ac.standrews.utils.main.setutils.Set


getSupersets() - Method in class uk.ac.standrews.utils.main.collectionutils.setutils.SupersetsManager


getSupersets() - Method in class uk.ac.standrews.utils.main.setutils.SupersetsManager


getSupersetsCSV() - Method in class uk.ac.standrews.utils.main.collectionutils.setutils.SupersetsManager
:   Get a comma separated list of [superset, subset] couples.

getSupersetsCSV() - Method in class uk.ac.standrews.utils.main.setutils.SupersetsManager
:   Get a comma separated list of [superset, subset] couples.

getSupersetsIds() - Method in class uk.ac.standrews.utils.main.collectionutils.setutils.SupersetsManager


getSupersetsIds() - Method in class uk.ac.standrews.utils.main.setutils.SupersetsManager


getSupersetsIdsCSV() - Method in class uk.ac.standrews.utils.main.collectionutils.setutils.SupersetsManager
:   Get a comma separated list of [superset id, subset id] couples.

getSupersetsIdsCSV() - Method in class uk.ac.standrews.utils.main.setutils.SupersetsManager
:   Get a comma separated list of [superset id, subset id] couples.

getTable() - Method in class uk.ac.standrews.utils.main.database.TableManager


getTableColumnsNames() - Method in class uk.ac.standrews.utils.main.database.TableReader
:   Gets the list of columns for a database table (from:
    http://forums.sun.com/thread.jspa?threadID=501222&messageID=2369725)

getTableCreator() - Method in class uk.ac.standrews.utils.main.database.Table


getTableName() - Method in class uk.ac.standrews.utils.main.database.Table
:   Get the table name

getTableName() - Method in class uk.ac.standrews.utils.main.database.TableManager


getTableReader() - Method in class uk.ac.standrews.utils.main.database.Table


getTableRow() - Method in class uk.ac.standrews.utils.main.database.Table


getTableRowsWhereEqual(String, String) - Method in class uk.ac.standrews.utils.main.database.TableReader
:   Gets the table rows where the given column has exactly the given value

getTableRowWhereEqual(String, String) - Method in class uk.ac.standrews.utils.main.database.TableReader
:   Gets the \*first\* table row where the given column has exactly the given
    value

getTableRowWhereLike(String, String) - Method in class uk.ac.standrews.utils.main.database.TableReader
:   Gets the \*first\* table row where the given column has a value similar to
    the given value

getTableWriter() - Method in class uk.ac.standrews.utils.main.database.Table


getTabSeparatedString(int[], String) - Static method in class uk.ac.standrews.utils.main.collectionutils.ArrayUtils
:   Returns a separator separated string representation for an array

getTagName() - Method in class uk.ac.standrews.utils.main.webutils.simpledomparser.XmlNode
:   Get the tag E.g.

getTest1Table() - Static method in class uk.ac.standrews.utils.test.database.TableTest


getTestDbManager() - Static method in class uk.ac.standrews.utils.test.database.DbManagerTest


getTestHtml() - Static method in class uk.ac.standrews.utils.test.RegExpUtilsTest


getText() - Method in class uk.ac.standrews.utils.main.webutils.simpledomparser.XmlNode
:   Get text for the tag E.g.

getTextFromChildElement(XmlNode, String, String, File) - Static method in class uk.ac.standrews.utils.main.webutils.simpledomparser.XmlSearcher
:   Checks if child exists and returns its text

getTimeDownloadedColumn() - Static method in class uk.ac.standrews.utils.main.database.DbUtils


getTimeOnlyStamp() - Static method in class uk.ac.standrews.utils.main.TimeUtils
:   Returns a time stamp in the format (for 11 am, 18 min, 47 sec): 111847
    hhmmss

getTimePassed(Calendar) - Static method in class uk.ac.standrews.utils.main.TimeUtils


getTimestampColumn() - Static method in class uk.ac.standrews.utils.main.database.DbUtils


getTimeStampForFileName() - Static method in class uk.ac.standrews.utils.main.TimeUtils
:   Date-time stamp (up to seconds) that can be attached to file names

getTruncatedPareto100() - Static method in class uk.ac.standrews.utils.test.stats.TruncatedParetoTest


getTruncationThreshold() - Method in class uk.ac.standrews.utils.main.stats.TruncatedPareto


getUniformRandomGenerator() - Method in class uk.ac.standrews.utils.main.stats.UniformRandomSingleton


getUniformRandomSubmap(Map, double) - Static method in class uk.ac.standrews.utils.main.stats.UniformRandomUtils
:   Returns a submap of randomly picked map entries

getUniqueValues() - Method in class uk.ac.standrews.utils.main.maputils.MapOfMaps
:   Get the set of unique values from all internal maps

getUrl() - Method in class uk.ac.standrews.utils.main.database.DbConn


getUser() - Method in class uk.ac.standrews.utils.main.database.DbConn


getValue(String) - Method in class uk.ac.standrews.utils.main.database.TableRow
:   Return the value for a certain column

getValue(Double) - Static method in class uk.ac.standrews.utils.main.NumberUtils
:   Gets a string value usable for SQL DOUBLE column.

getValues() - Method in class uk.ac.standrews.utils.main.database.TableRow


getValues() - Method in class uk.ac.standrews.utils.main.maputils.OneToManyMap
:   Get the set of unique values for this map.

getValues() - Method in class uk.ac.standrews.utils.main.maputils.TableMap


getValuesStringPool() - Method in class uk.ac.standrews.utils.main.maputils.MapOfMaps


getValuesStrings(Properties) - Static method in class uk.ac.standrews.utils.main.PropertiesUtils
:   Get the properties values as strings

getWriter() - Static method in class uk.ac.standrews.utils.test.database.TableWriterTest


getXml() - Static method in class uk.ac.standrews.utils.test.XmlUtilsTest


getXml1String() - Static method in class uk.ac.standrews.utils.test.XmlNodeTest


getXml2String() - Static method in class uk.ac.standrews.utils.test.XmlNodeTest


getXml3String() - Static method in class uk.ac.standrews.utils.test.XmlNodeTest


getXml4String() - Static method in class uk.ac.standrews.utils.test.XmlNodeTest


getXml5String() - Static method in class uk.ac.standrews.utils.test.XmlNodeTest


getXml6String() - Static method in class uk.ac.standrews.utils.test.XmlNodeTest


getXml7String() - Static method in class uk.ac.standrews.utils.test.XmlNodeTest


getXmlComment(String) - Static method in class uk.ac.standrews.utils.main.webutils.XMLUtils
:   Returns a string formatted as xml comment:

getXmlFromHTML(String, String) - Static method in class uk.ac.standrews.utils.main.webutils.XMLUtils


getXmlFromReader(BufferedReader) - Static method in class uk.ac.standrews.utils.main.webutils.XMLUtils
:   Get the XML as SimpleElement from a URL reader.

getXmlString() - Static method in class uk.ac.standrews.utils.test.XmlNodeTest


getXmlStringReader() - Static method in class uk.ac.standrews.utils.test.XmlUtilsTest


getXmlTreeFromString(String) - Static method in class uk.ac.standrews.utils.main.webutils.simpledomparser.XmlNode


getXmlTreeFromUrl(String, boolean) - Static method in class uk.ac.standrews.utils.main.webutils.simpledomparser.XmlNode
:   get XML tree from URL

GuiUtils - Class in uk.ac.standrews.utils.main.guiutils
:   Gui utilities

GuiUtils() - Constructor for class uk.ac.standrews.utils.main.guiutils.GuiUtils


gunzipFile(File) - Static method in class uk.ac.standrews.utils.main.FileUtils


gunzipFile2(File, File) - Static method in class uk.ac.standrews.utils.main.FileUtils
:   Gunzip the input archive.

A B C D E F G H I J K L M N O P Q R S T U V W X Y

- Overview
- Package
- Class
- Use
- Tree
- Deprecated
- Index
- Help

- Prev Letter
- Next Letter

- Frames
- No Frames

- All Classes
